# Lower limb salvage in necrotizing burn wound infection: The role of fibular ostectomy and local flaps in a resource-limited setting – A case report and literature review

**DOI:** 10.1016/j.ijscr.2024.110054

**Published:** 2024-07-18

**Authors:** Anteneh Meaza Dawit

**Affiliations:** Addis Ababa University, Department of Plastic & Reconstructive surgery, Resident physician, Ethiopia

**Keywords:** Necrotizing soft tissue infection, Burn wound infection, Fibular ostectomy, Bipedicle flap, Microsurgical free tissue transfer

## Abstract

**Introduction:**

Necrotizing burn wound infections following burn injuries are rare. Literature on these cases is also scarce. These infections are life- and limb- threatening unless properly managed. They also pose significant reconstructive challenge, especially in settings lacking microvascular capability. This report describes a limb preservation strategy for limb-threatening necrotizing infection of the leg that complicated a burn injury. Innovative approach was used, utilizing proximal fibular ostectomy, bipedicled local advancement flap and split thickness skin graft.

**Case presentation:**

A 26-year-old female patient presented to our burn unit after sustaining a contact burn injury from a burning charcoal to her right lateral leg within three days. On the second day of admission, the patient developed significant changes in the appearance of the wound, leading to the diagnosis of necrotizing myofacitis. Emergent debridements were done with the aim of preserving the limb. Subsequent successful, albeit sub-optimal, reconstruction was also achieved despite the lack of microvascular surgical capability in the burn unit.

**Discussion:**

This case report and literature review describes a rare limb-threatening necrotizing burn wound infection. The significant reconstructive challenge posed by the defect was addressed using a simple but rarely described reconstructive technique. The importance of limb preservation in LMIC is also emphasized.

**Conclusion:**

The goal of preserving a limb can be met by using a simple reconstructive technique, despite the lack of microvascular capabilities.

## Highlights

• This report describes a rare limb- threatening necrotizing burn wound infection managed in a resource limited setting.
• The report presents utilization of proximal fibular ostectomy and local flap for lower limb preservation.

## 1 Introduction

Necrotizing burn wound infections following burn injuries are rare. Literature on this cases is also scarce. This type of infections are life and limb threatening unless properly managed. They also pose a significant reconstructive challenge for limb preservation.

This report describes a rare limb threatening necrotizing infection of the leg that complicated a burn injury. A technique using proximal fibular ostectomy, bipedicled transposition local flap and STSG is also described. This simple but rarely described technique was crucial in preserving the limb of the patient who otherwise would have ended up in an amputation. This report adheres to the SCARE criteria [1].

## 2 Case presentation

A 26-year-old female patient presented to our burn unit after sustaining a contact burn injury from a burning charcoal to her right lateral leg within three days. The patient was in a confined small room during the incident and she had a loss of consciousness up on recovery by her family members.

**Fig. 1:**
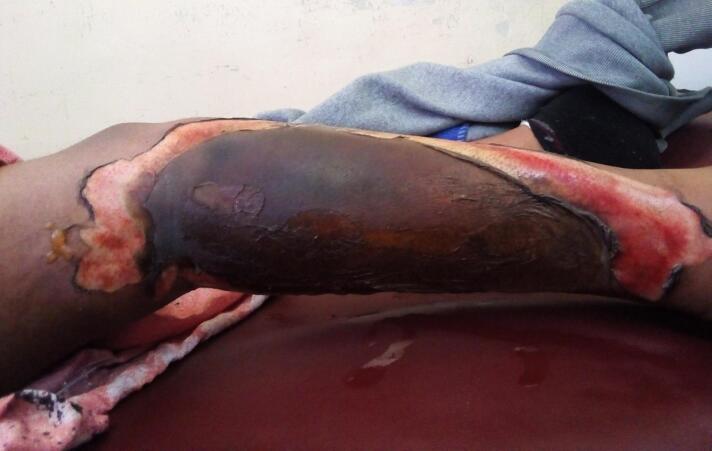
Appearance of the wound up on initial presentation demonstrating a full thickness burn wound over the right lateral leg.

Up on initial presentation she was having normal vital signs. Physical examination revealed a full thickness burn wound over the lateral right leg (Fig. 1) extending from the knee joint to lateral ankle area. All blood workups were in normal range (Table 1). Subsequently with a diagnosis of 7 % TBSA full thickness burn injury she was admitted to the burn unit and was started on daily dressing changes, nutritional management and analgesia and was waiting for elective excision and STSG.

**Table 1:** Investigation profile of the patient on the day of admission and on subsequent follow-up.

Admission	3rd follow-up day
CBC	CBC
WBC = 12.4 (*N* = 78 %, L = 19 %) Hb = 12.1 g/dL, HCT = 35 % MCV = 95 fL, MCH = 34 pg PLT = 166	WBC = 14.7 (*N* = 93 %, L = 4.9 %) Hb = 9.2 g/dL, HCT = 35 % MCV = 95 fL, MCH = 34 pg PLT = 162

OFT and Electrolytes	OFT and Electrolytes
ALT = 47, AST = 207, SCr = 0.31 mg/dL Electrolytes = In normal range	ALT = 45, AST = 210, SCr = 0.4 mg/dL Electrolytes = In normal range

**Fig. 2:**
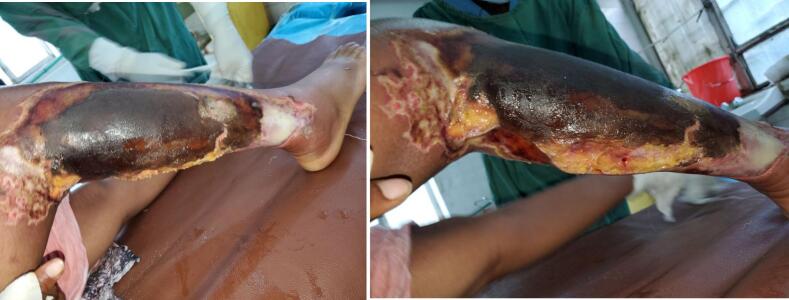
Appearance of the right leg post burn wound on 3rd day demonstrating significant necrosis and edema.

On the second day of admission the patient developed worsening of pain and a foul smelling watery discharge over the wound area. There was also subjective fever, loss of appetite and malaise. Physical examination revealed significant swelling and erythema over the surrounding skin (Fig. 2) There was also significant tenderness but no crepitus was present. Updated investigations showed leukocytosis with left shift of neutrophil = 93 % (Table 1).

**Fig. 3:**
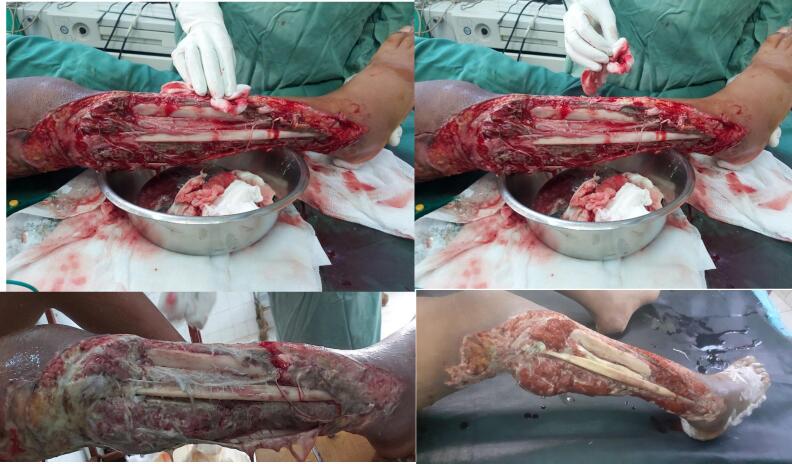
Intraoperative picture showing initial radical debridement that left the leg bones exposed with and subsequent follow-up pictures of the defect demonstrating significant necrotic tissue.

With the diagnosis of necrotizing soft tissue infection, the patient was started on Ceftriaxone 1 g IV BID, Metronidazole 500 mg IV TID and Vancomycin 1 g IV TID and was taken to OR for radical debridement. Intraoperative finding revealed a foul smelling turbid fluid with associated necrotic anterior and lateral compartment muscles (Fig. 3). There was also a partial necrosis over the superficial posterior compartment muscle settling the diagnosis of necrotizing myofacitis. Microbiologic investigations were not done due to limited capability of the setup.

Repeat debridements were made on the following days leaving the fibula and anterio-lateral cortex of tibia completely exposed. Wound care was continued with the aim of preserving the limb. Topical silver sulfadiazine antibiotic dressing on iodine gauze were used in addition to systemic antibiotics to control the infection.

**Fig. 4:**
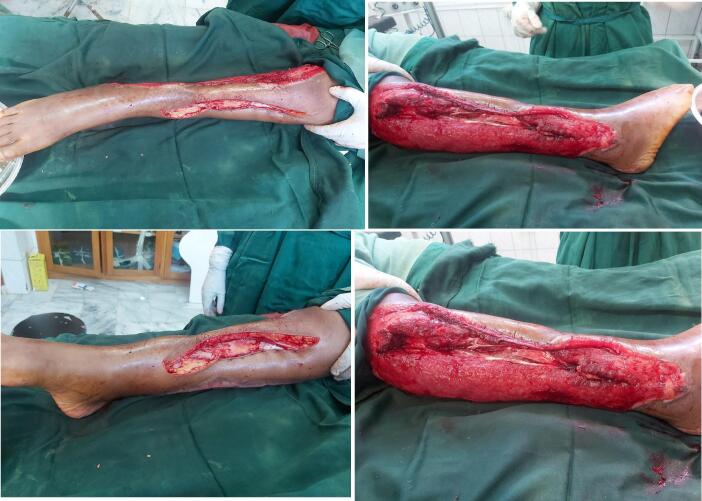
Intraoperative pictures demonstrating the right leg defect after proximal fibulectomy and bipedicled flap reconstruction.

**Fig. 5:**
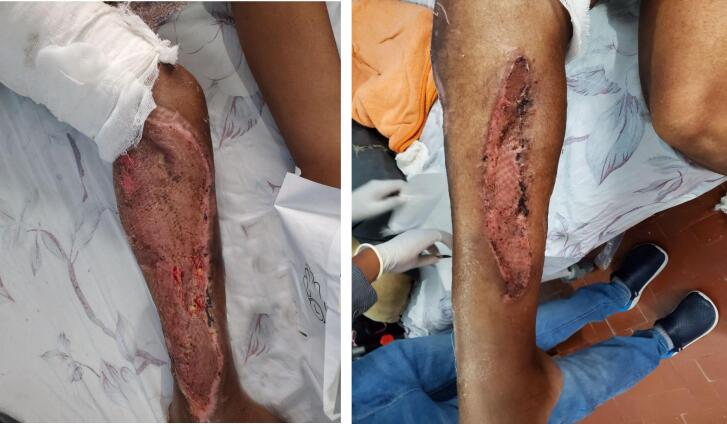
Final appearance of the right leg with good graft take.

After controlling the infection reconstructive plan was made. Due to the limited capability to do free flap in the burn unit, proximal fibular ostectomy was done preserving its periosteum and the distal 6 cm of the bone for ankle joint stability. Anterolateral cortex of tibia was chiseled to remove the desiccated cortical bone until a grossly healthy bleeding bone was encountered. This was followed by a bi-pedicled transposition flap from the medial leg that was advanced laterally to cover the exposed tibia (Fig. 4). The rest of granulating wound and the secondary defect was covered by a meshed STSG (Fig. 5).

The patient underwent a total of 5 operations, required 6 U of blood and stayed for 2 months in the unit. Despite being time and resource consuming limb salvage was achieved without the use of free flaps. The patient was finally discharged from the ward after good take of the graft with a POP cast splint. She is currently on follow-up for the foot drop secondary to common perioneal nerve injury and is in need of subsequent managements.

## 3 Discussion

Necrotizing soft tissue infections following burn injuries are infrequent [2,3], and reports are scarce. Those that are reported followed scaled and contact burns [[2], [3], [4]]. Necrotizing fasciitis in a post burn contracture scar has also been reported [4]. Symptoms of NF include rapid progression of symptoms with disproportionate pain, fever and signs of sepsis [[2], [3], [4]], as seen in this patient. Marked tissue edema, crepitus, ecchymosis, and bullous skin changes are also commonly seen.

The inflammatory signs that normally accompany burns makes the diagnosis challenging in burn injury patients. Although there is a clinical prediction tool (LRINEC score) to diagnose NF its sensitivity is low at 68.2 % and its effectiveness in burn injuries is not well established. Therefore, the diagnosis of NF should be made based entirely upon clinical suspicion [5].

Once clinically diagnosed the management should be prompt and aggressive to prevent mortality and limb loss [2,3]. Patients should be resuscitated and blood workup ordered. Empiric broad spectrum antimicrobials should be initiated taking in to account that NF is a polymicrobial infection. Blood should be prepared for possible transfusion and aggressive initial debridement should be made. Antibiotic alone is insufficient and should always be accompanied by surgical debridement as systemic antibiotics poorly reach the necrotic tissue [3].

Delay in initiation of surgical management and a less aggressive initial debridement can result in amputation [2]. And even with appropriate management the mortality rate in this group of patients is high reaching 20–40 % [6,7] implying the need for aggressive surgical debridement as a cornerstone of successful management.

The diagnosis of NF can be made intraoperatively by the presence of “dishwater fluid” and “positive finger sign” which is a lack of resistance during dissection in to tissue planes [6]. These signs were also seen in this patient.

The only risk factors for NF in this patient were, the burn insult which causes immunosuppression and the burn wound environment which has a necrotic tissue and protein–rich exudate that provides a suitable growth medium for bacteria [8]. Other predisposing chronic illnesses were lacking.

The surgical management was done by repeated radical debridements followed by reconstruction. In other patients limb amputation may be the only viable or lifesaving option of management.

Limb amputation is a major undertaking that leads to permanent disability. It has a profound negative impact on an individual's functionality and ability to perform activities of daily living [8,9]. The procedure itself carries high rate of surgical complications. In addition, the rehabilitation can be protracted. In LMIC where functional prosthesis is extremely limited in availability the final functional outcome after amputation is also quite poor [10].

Several factors should be considered before deciding to do amputation including the salvagablity of the limb, demographics, economic status and local availability of a sustainable reliable rehabilitation programs [9,10].

Deciding on the type of limbs that should be amputated or those that can be salvaged is a matter of controversy. There are several lower limb injury severity scoring systems to guide this decision. However, this scoring systems have not been shown to be a good predictors of limb amputation or salvage. Rather the final outcomes depend on the surgeons' experience and capabilities of the setups [11].

In low resource setups with weak rehabilitation programs where the socioeconomic and physical outcomes after lower limb amputation are believed to be suboptimal limb preservation should be a main goal [11]. Every effort to salvage the limb should be pursued and its outcome can be rewarding [11].

Considering the fact that this patient is a relatively young female patient in a low income country with limited access to prosthesis [10] and expected to have a poor outcome after LEA [9] a decision was made to preserve the limb. As seen in this patient's final outcome, limb salvage can be achieved even in adverse working conditions and the outcome can be rewarding [11].

Limb preservation is not without its own problems however and has considerable morbidity [3,12]. It leads to multiple surgeries longer duration of hospital stay and poses a wide defect that is a complex surgical challenge [[12], [13], [14]]. This difficulty is associated with the limited regional soft tissue availability, especially for the lower third of the leg [13].

Prolonged cortical bone exposure can result in bone desiccation and necrosis leading to osteomyelitis [14] necessitating urgent bone coverage. The specific time limit at which there can be satisfactory bone coverage after exposure is not well established but successful coverage has been reported even after 5 months of exposure with good outcome [14].

The possible surgical strategies for coverage of this type of defects include: microvascular free tissue transfer, use of NPWT combined with dermal substitutes and use of local or regional flaps [14].

**Microsurgical free tissue transfer (Free flap)** reconstruction would provide a solid and instant coverage for the exposed bone [14]. Free flap has become a first choice treatment and sometimes a last resort for limb salvage. The success rate of free tissue transfer in lower leg defects is 80–100 % but can be as low as 73 % when reconstruction is delayed after initial trauma [14]. The lack of capability to do free flap in the burn unit obviated its use. Referral to another hospital with capability to do free flaps wasn't also an option due to lack of available free bed at the time.

In situations where free flap is not an option for any sort of reason another alternative is use of NPWT, possibly preceded by drilling of the exposed tibia and use of bioengineered, cell-free dermal matrix or dermal substitutes (Glyaderm or integra) [[14], [15], [16]]. This can create a stable granulation tissue that can be skin grafted [14].

**Bone drilling and NPWT** are commonly used in cases with limited bone exposure. The process is protracted, with granulation formation often being limited to metaphysis and very slow on the diaphysis [13]. In addition, it doesn't yield an optimum result [13]. For this patient dermal substitutes were not available and the only reliable option was the use of local or regional flaps.

There are several options of regional and local flaps. This includes: Cross leg flap, muscle flaps with STSG, and bipedicled flap.

**Cross leg flap:** is a fasciocutaneous flap from the contralateral leg. It is a reliable flap but has its own drawbacks. It is a morbid procedure that causes large defect on the donor that also has to be skin grafted. It also needs immobilization Ex-Fix frame for 3-4 weeks risking joint stiffness and thromboembolism [11]. And despite consideration, the patient did not allow the other leg to be subjected to surgery, so it was obviated as an option.

**Muscle flaps:** Although uncommon in practice, the concominent use of three local muscle flaps has also been reported. The procedure involves the concomitant use of three muscle flaps including the medial gastrocnemius, tibialis anterior transposition and hemisoleous muscles [13]. The complications reported with this procedure include flap failure, Chronic lymphedema and chronic osteomyelitis [13]. The issue in this patient was that, the tibialis anterior had already been completely debrided and successful coverage of the tibia couldn't be achieved with this strategy.

**Bipedicled flap:** A successful procedure was finally achieved by performing partial proximal fibular ostectomy and bipedicled transposition flap of medial leg skin to cover the exposed bone. A bipedicled flap is a random skin flaps which gets blood supply from both ends. Its use in reconstructing lower leg defects is well described, with higher chance of flap survival compared to free flaps (100 % Vs 93 %) [17]. It has a short operation time, low cost and minimal donor site morbidity. In addition, it is easier to perform [18,19]. In some reports its considered an alternative option to free flap for coverage of lower leg defects [20].

The limited size of the bipedicled flap combined with the fact that the fibula was desiccated led to the decision to do partial fibular excision. This helped to better advance the flap laterally. The secondary and remaining primary defects were subsequently skin graftted. The upper fibula being an expendable bone [21,22] allowed for a relatively safe ostectomy (upper partial fibulectomy/ proximal fibulectomy). The use of this procedure is well established in several other pathologies including early knee osteoarthritis [23] and benign and malignant tumors of the fibula and hasn't been shown to have any side effects [22].

Fibula is a supportive bone that has minimal weight-bearing capacity. Only its distal articulation which stabilizes the ankle joint must be preserved. Proximal fibular resection is overall a difficult operation due to the anatomy of this location, particularly with the trifurcation of popliteal artery. The most critical structure is the posterior tibial vessel along with the tibial nerve and has to be preserved[21,22]. Common complications associated with fibular excision include injury to trifurcation of vessels and common perionial nerve [22].

In this patient, the common peroneal nerve was believed to be debrided during the initial surgery and the patient already had a foot drop prior to the reconstruction. The long-term gait outcome after proximal fibulectomy also remains to be seen in this patient.

## 4 Conclusion

In conclusion, the goal of achieving a supportive limb can be met by using this simple but rarely utilized reconstructive techniques. In LMIC with poor rehabilitation programs limb salvage should be a perimary goal and when microsurgical services are not available, fibular ostectomy and bipedicled flap reconstruction can serve as a potential limb salvage strategies. Further research is also needed to assess the long term outcome of patients undergoing this type of limb reconstruction.

## Consent

Written informed consent was obtained from the patient for publication and any accompanying images. A copy of the written consent is available for review by the Editor-in-Chief of this journal on request.

## Ethical approval

No ethical committee clearance or approval was needed to write a case report in my institution.

## Funding

This report did not receive any form of funding from any funding agencies in the public, commercial, or not-for-profit sectors.

## Author contribution

The author was responsible for the patient management and drafting, editing and writing of the manuscript.

## Guarantor

Anteneh Meaza Dawit.

## Research registration number

N/A.

## Conflict of interest statement

No conflict of interest has affected this report and review.
